# *Trichuris trichiura* infection and its relation to environmental factors in Mbeya region, Tanzania: A cross-sectional, population-based study

**DOI:** 10.1371/journal.pone.0175137

**Published:** 2017-04-06

**Authors:** Kirsi M. Manz, Petra Clowes, Inge Kroidl, Dickens O. Kowuor, Christof Geldmacher, Nyanda E. Ntinginya, Leonard Maboko, Michael Hoelscher, Elmar Saathoff

**Affiliations:** 1 Institute for Medical Informatics, Biometry and Epidemiology, Ludwig-Maximilians-University, Munich, Germany; 2 Division of Infectious Diseases and Tropical Medicine, Medical Center of the University of Munich (LMU), Munich, Germany; 3 NIMR-Mbeya Medical Research Center (MMRC), Mbeya, Tanzania; 4 German Center for Infection Research (DZIF), partner site Munich, Munich, Germany; George Washington University School of Medicine and Health Sciences, UNITED STATES

## Abstract

**Background:**

The intestinal nematode *Trichuris trichiura* is among the most common causes of human infectious disease worldwide. As for other soil-transmitted nematodes, its reproductive success and thus prevalence and intensity of infection in a given area strongly depend on environmental conditions. Characterization of the influence of environmental factors can therefore aid to identify infection hot spots for targeted mass treatment.

**Methodology:**

We analyzed data from a cross-sectional survey including 6234 participants from nine distinct study sites in Mbeya region, Tanzania. A geographic information system was used to combine remotely sensed and individual data, which were analyzed using uni- and multivariable Poisson regression. Household clustering was accounted for and when necessary, fractional polynomials were used to capture non-linear relationships between *T. trichiura* infection prevalence and environmental variables.

**Principal findings:**

*T*. *trichiura* infection was restricted to the Kyela site, close to Lake Nyasa with only very few cases in the other eight sites. The prevalence of *T*. *trichiura* infection in Kyela was 26.6% (95% confidence interval (CI) 23.9 to 29.6%). Multivariable models revealed a positive association of infection with denser vegetation (prevalence ratio (PR) per 0.1 EVI units = 2.12, CI 1.28 to 3.50) and inverse associations with rainfall (PR per 100 mm = 0.54, CI 0.44 to 0.67) and elevation (PR per meter = 0.89, CI 0.86 to 0.93) while adjusting for age and previous worm treatment. Slope of the terrain was modelled non-linearly and also showed a positive association with *T*. *trichiura* infection (p-value p<0.001).

**Conclusion/Significance:**

Higher prevalences of *T. trichiura* infection were only found in Kyela, a study site characterized by denser vegetation, high rainfall, low elevation and flat terrain. But even within this site, we found significant influences of vegetation density, rainfall, elevation and slope on *T. trichiura* infection. The inverse association of rainfall with infection in Kyela is likely due to the fact, that rainfall in this site is beyond the optimum conditions for egg development. Our findings demonstrate that use of remotely sensed environmental data can aid to predict high-risk areas for targeted helminth control.

## Introduction

Infections with soil-transmitted helminths, a group of nematodes affecting humans, are among the most common infections worldwide. The most common helminth species are the roundworm (*Ascaris lumbricoides*), the whipworm (*Trichuris trichiura*) and the hookworms (the two species *Ancylostoma duodenale* and *Necator americanus*). *T*. *trichiura* infection can cause diarrhea, malnutrition, growth retardation and anemia, but light infections are commonly asymptomatic [1]. According to recent estimates, *T*. *trichiura* accounts for about 465 million infections world-wide [2]. Sub-Saharan Africa is one of the regions still heavily affected by soil-transmitted helminth infections, since their transmission is enhanced by poor hygienic conditions and poverty. Unfortunately, despite the efforts of preventive mass chemotherapy conducted in many sub-Saharan African countries [3], the *T*. *trichiura* prevalence there has not recently declined [2, 4]. Indeed, *T*. *trichiura* infection seems to be difficult to cure, since the available drugs are not very effective against this helminth infection [5, 6].

*T*. *trichiura* infection occurs after ingestion of embryonated eggs from the soil [7]. The eggs hatch inside the human intestine and release larvae. The larvae mature and the adult females living in the small intestine begin to produce eggs. The eggs are excreted together with the feces and undergo embryonation, the temperature-dependent development to the infective stage [8]. During the development in the soil, the eggs are exposed to environmental factors such as rain, soil humidity, and soil temperature, which can favor or hinder their development. For *T*. *trichiura* eggs the upper temperature limit for survival is about 37–38°C. Beyond this threshold, the eggs will not develop to the infective stage [1, 8].

Gaining knowledge about the factors contributing to *T*. *trichiura* infection is of great interest. A promising low cost approach for predicting the infection prevalence is the use of remote sensing data and geographical information systems [9–11]. Remotely sensed satellite data can be used to obtain environmental information for wide areas. A satellite detects electromagnetic radiation reflected from the surface of the earth. This radiation can be used to characterize environmental conditions on the ground such as temperature or green vegetation cover. A great advantage of this approach is the availability of these data in the public domain. Linking environmental data with *T*. *trichiura* infection allows comparing ecological data with individual disease status. This can be helpful in identifying environmental conditions associated with the infection, which can in turn be used to characterize high-risk areas for targeted worm control programs.

No prevalence estimates for *T*. *trichiura* infection in southwestern Tanzania are available from the literature [12]. This study provides new information on the prevalence and the spatial distribution of *T*. *trichiura* infection from an epidemiological survey conducted in Mbeya region in southwestern Tanzania. Our aim was to investigate the associations of satellite derived environmental data with *T*. *trichiura* infection while considering the effect of potential confounders, such as age, sex and socio-economic status.

## Methods

### Ethics statement

This study was approved by the ethics committee of the Tanzanian National Institute for Medical Research and the Mbeya Medical Research and Ethics Committee and conducted in accordance with the Declaration of Helsinki. All adult participants provided written informed consent before enrollment into the study with parents consenting for their minor children, who were in addition asked for their assent if above 12 years of age.

### Study area and epidemiological data collection

**Fig 1:**
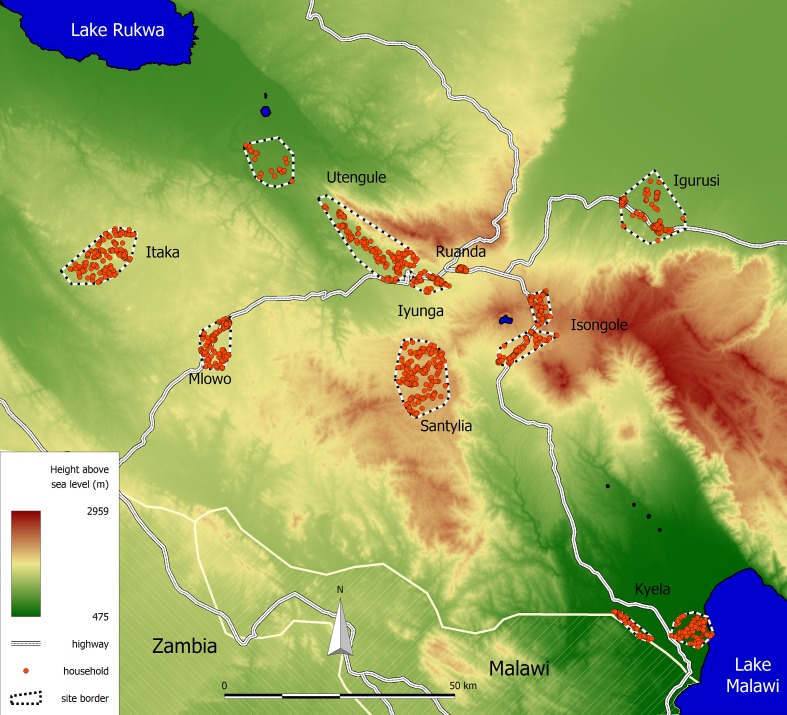
Location of the EMINI study area. The study area consists of nine distinct study sites. Participating household are shown as red circles.

The study area is located in the Mbeya region in southwestern Tanzania and extends from 32.678° to 33.963° East and from 8.652° to 9.649° South. An overview of the study area, the study sites and the participating households is shown in Fig 1. The data was collected between June 2008 and June 2009 as a part of the third annual survey of the EMINI (Evaluating and Monitoring the Impact of New Interventions) cohort study. Below we briefly summarize data collection procedures for this study. A more detailed description of the study area and data collection is provided in Riess *et al*. [13].

Initially nine different study sites in Mbeya region were chosen to represent a wide variety of environmental and economic conditions. After an initial census covering more than 42,000 households from these nine sites, a geographically stratified random sample of 10% of the households was chosen to participate in the EMINI study. Each household’s position was determined using handheld GPS devices. During each annual visit, all household members were asked for blood and urine samples and interviewed in Kiswahili language and their answers recorded using handheld computers. Additionally, we collected stool samples from the third annual survey in 2008 onwards from 50% of all households. In our present analysis, we made use of the data from this third survey. The participant helminth infection status was not assessed previously and to our knowledge, there were no other helminth treatment programs conducted in the region. This allows us to capture the natural prevalence of the infection, which is not affected by any running programs.

Upon collection, stool samples were refrigerated using mobile refrigerators and kept cool until slide preparation within two days after specimen collection. The presence of *T*. *trichiura* infection was established by Kato-Katz examination of two 41.7 mg subsamples from a single stool specimen and defined as existence of at least one *T*. *trichiura* egg in any of the two stool slides. The slides were examined by experienced staff within two days after slide preparation. Participants with helminth infection were offered treatment with albendazole (for *T*. *trichiura* and other intestinal nematode infections) and/or praziquantel (for schistosome infections).

Socio-economic status (SES) can be a potential confounder and should thus be assessed. In low resource settings, information on household income and expenses often fails to characterize the socio-economic status correctly, since non-cash income also plays an important role. A modified method initially proposed by Filmer and Pritchett [14, 15] was used to characterize the socio-economic situation of each household. This method uses principal component analysis to generate an SES score using proxy variables for household wealth. For our study, the SES score was constructed from household belongings (clock or watch, radio, television, mobile telephone, refrigerator, hand cart, bicycle, motor cycle, car, savings account), materials used to build the house, sources of energy and drinking water, number of persons per room and availability and type of latrine. This information was obtained from the head of the household and by direct observation during the interviews.

### Ecological data

Elevation data for our study were retrieved from the NASA Shuttle Radar Topography Mission (SRTM) global digital elevation model, version 2.1. [16]. These data were also used to calculate the slope of the terrain. Land surface temperature during the day (LST day) and night (LST night), and green vegetation cover (EVI = enhanced vegetation index) data were collected from NASA’s Moderate-Resolution Imaging Spectroradiometer (MODIS) Terra satellite during 2003 to 2008. These data were retrieved from the online data pool, courtesy of the NASA EOSDIS Land Processes Distributed Active Archive Center (LP DAAC), USGS/Earth Resources Observation and Science (EROS) Center, Sioux Falls, South Dakota (https://lpdaac.usgs.gov/) [17] and were used to produce long-term averages of LST day, LST night and EVI, as previously described [13]. Mean annual rainfall was obtained from the WorldClim—Global Climate Data website (http://www.worldclim.org/).

Household positions and number of inhabitants were known from the initial population census and used to calculate population densities. The population density, LST, EVI, rainfall and elevation data were then averaged for a buffer area of 1000 m radius around each household to characterize the situation around the household.

### Statistical analyses

All statistical analyses were performed using Stata Statistical Software (Release 14. College Station, TX: StataCorp LP). Age, sex, socio-economic status, population density, latrine coverage in the surroundings, presence of a latrine in the household and previous worm treatment were included into our analyses as potential confounders.

Since environmental data is prone to be highly correlated, multicollinearity of independent variables was assessed using the variance inflation factor (VIF). Variables with a VIF of 10 or above were regarded as seriously collinear [18, 19] and not entered simultaneously into the same multivariable model.

Due to only light to moderate *T*. *trichiura* infection intensity and thus low egg counts we modelled the infection outcome as a binary variable (negative vs. positive). We applied the following variable transformations to improve interpretability of results: the reported prevalence ratios (PRs) correspond to an increase of 1000 persons/km^2^ for the population density, 0.1 units for mean annual EVI, 0.1°C for mean annual LST night, 100 mm for rainfall and 10% for latrine prevalence in the surroundings. Age was categorized in groups of 0 to 5 years, 5 to 20 years and above 20 years, since a non-linear relationship of *T*. *trichiura* infection and age was expected from the literature [1].

To assess the association of different factors with *T*. *trichiura* infection we used Poisson regression with robust (or Huber/White or Sandwich) variance estimates because we wanted to estimate prevalence ratios that are more intuitively interpretable for common outcomes than odds ratios. The standard regression approach to estimate risk and prevalence ratios is the log-binomial model [20], however, if log-binomial models do not converge, which was the case for some of our models, Poisson regression with robust variance estimates is a valid alternative [21, 22]. At first, we performed univariable Poisson regression to estimate prevalence ratios of *T*. *trichiura* infection and their 95% confidence intervals. Since individual observations from the same household tend to be dependent of each other, our data is clustered within households. To account for this, robust standard errors adjusted for household clustering were calculated. After each univariable analysis step we performed a non-linearity test for the respective independent variable [23]. The test splits the continuous variable into ten bins, refits the model and performs an overall Wald test with significant test results indicating non-linearity.

All variables with univariable p-values below 0.2 were initially included in the multivariable analysis. Multivariable Poisson regression was performed by starting with a model including only individual level covariates such as sex, age and worm treatment history. The model was extended by including household level covariates such as availability of latrine or socio-economic status. As a third step, we included environmental variables one by one. Variables were retained in the model if their p-values stayed < = 0.05 and variables with p-values above 0.05 were removed. Decreases in Akaike Information Criterion (AIC) and Bayesian Information Criterion (BIC) indicated better model fit and were used as additional information for inclusion of new variables.

Because of the non-linearity of the environmental data, we also used fractional polynomials modelling to check, if the multivariable linear Poisson models required inclusion of non-linear terms. This approach detects non-linear associations by fitting a predefined set of polynomials to the data and comparing the deviance of the models [24, 25]. The procedure is only carried forward if the non-linear data fit the model better than linear data. Otherwise, the procedure is stopped and the original linear variable is used. A p-value threshold of < = 0.01 was chosen for the function selection procedure to avoid overfitting.

Spatial autocorrelation refers to situations where neighboring observations tend to be more similar than observations further apart. This poses a problem, since the central assumption of regression techniques, the independence of observations is violated. In our study setting, spatial autocorrelation is present, when the probability to be infected with *T*. *trichiura* depends on the location where the participant lives. To assess the existence and degree of spatial autocorrelation, Moran’s I [26] was calculated for different distances between household positions. The deviance residuals of our final models were plotted together with Moran’s I, to assess if the model reduced the spatial autocorrelation that was present in the raw data.

## Results

### Descriptive statistics

**Fig 2:**
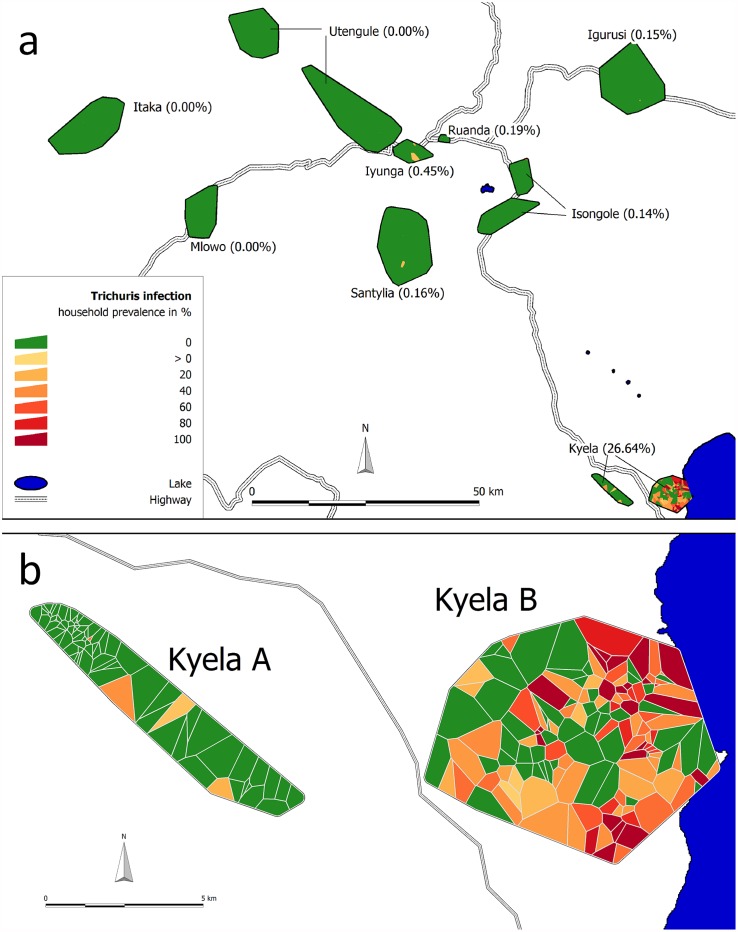
Prevalence of *T*. *trichiura* infection in the nine EMINI study sites in Mbeya region, Tanzania (A) and details for Kyela site (B). Households with at least one infected person are represented by red Voronoi polygons, households without are shown in green. Subsite A and B in this text refer to the western and eastern part of Kyela, respectively.

**Fig 3:**
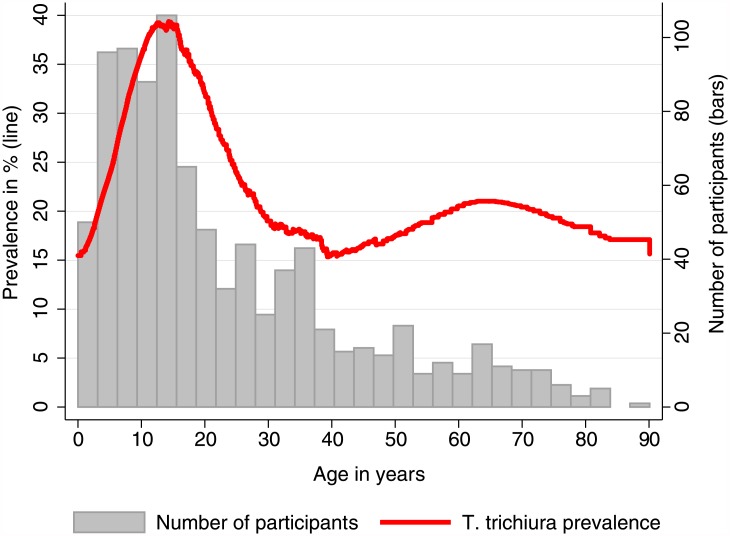
*T*. *trichiura* prevalence by age. The red line shows LOWESS-smoothed *T*. *trichiura* infection prevalence, grey bars indicate the number of participants in each age stratum.

Data for 6234 participants (from 1,610 different households) were available for this analysis. The median age of the study population was 17 years (interquartile range (IQR) 9 to 35 years) and 53% (3306/6234) were females. The overall prevalence of *T*. *trichiura* infection in all nine sites was 4.0% (249/6234, 95% confidence interval (CI) 3.5 to 4.5%). We found a unique local prevalence maximum of 26.6% (243/912, CI 23.9 to 29.6%) in Kyela study site (Table 1). In other sites, only isolated *T*. *trichiura* infections were found with two or less cases per site (Fig 2). Since it is likely that these infections had been acquired elsewhere and not within the sites, we only included the high-prevalence site Kyela in our further analyses. Kyela site is divided in two subsites, Kyela A in the west and Kyela B which is further to the east and closer to Lake Nyasa (Fig 2). The main environmental differences between Kyela A and B (see also Table 1) are that Kyela B is lower located and flat and the temperature variations between day and night are less than in Kyela A. In addition to this, the participants of Kyela B site have less latrines and lower socio-economic status, factors potentially favoring the spread of the worm infection. While in Kyela A only 8 (2.7%, CI 1.4 to 5.3%) participants were infected with *T*. *trichiura*, the number of infected participants in Kyela B was 235 (38.1%, CI 34.3 to 42.0%). Infections were mostly of light intensity and no heavy infections were found [27]. The maximum prevalence of *T*. *trichiura* infection occurred before the age of 15 years (Fig 3) and decreased after that. A second, much less prominent peak occurred at higher ages.

**Table 1:** Characteristics of the study population and environmental conditions at their place of residence.

Variable Unit		Kyela A	Kyela B	Kyela overall	Other sites
**Number of observations**	N	295	617	912	5,322
**Age (years)**	Median	15.1	16.8	16.2	16.8
	(IQR)	(8.7–31.8)	(9.0–35.1)	(8.9–33.8)	(8.9–35.7)
**Sex male**	%	50.2	48.5	49.0	46.6
***T*. *trichiura* infection prevalence**	%	2.7	38.1	26.6	0.1
**Infection intensity**^a)^**:**					
**No infection (0 EPG)**	%	97.3	61.9	73.4	99.9
**Light infection (1–999 EPG)**	%	2.7	36.6	25.7	0.1
**Moderate infection (1000–9999 EPG)**	%	0.0	1.5	1.0	0.0
**Heavy infection (≥10000 EPG)**	%	0.0	0.0	0.0	0.0
**Co-infection with *A*. *lumbricoides* and/or hookworm**^b)^	n (%)	7 (87.5)	186 (79.2)	193 (79.4)	1 (16.7)
**SES score**	Median	0.08	-0.73	-0.60	-0.01
	(IQR)	(-0.76–0.38)	(-1.21 –-0.21)	(-1.10–0.10)	(-0.52–0.61)
**Latrine coverage in surroundings (%)**	Median	99.3	92.1	96.4	100.0
	(IQR)	(98.2–100.0)	(76.6–97.3)	(83.8–99.1)	(98.8–100.0)
**Worm treatment last year**					
**Treated**	%	4.1	4.1	4.1	6.9
**Untreated**	%	64.8	60.6	62.0	88.9
**No information**	%	31.2	35.3	34.0	4.2
**Latrine type**					
**None**	%	2.0	11.8	8.7	1.5
**Simple pit latrine**	%	94.2	88.2	90.1	92.1
**Ventilated pit latrine**	%	2.0	0.0	0.7	4.2
**Water flush toilet**	%	1.7	0.0	0.6	2.2
**Population density (persons/km^2^)**	Median	1,117	423	441	404
	(IQR)	(285–2,326)	(340–520)	(319–644)	(207–2,348)
**Household density (households/ km^2^)**	Median	316	110	111	99
	(IQR)	(70–662)	(87–142)	(80–171)	(48–593)
**EVI**	Median	0.33	0.39	0.37	0.28
	(IQR)	(0.32–0.34)	(0.37–0.41)	(0.34–0.40)	(0.25–0.31)
**LST Day (°C)**	Median	33.6	31.0	32.1	33.4
	(IQR)	(33.2–33.9)	(29.9–32.1)	(30.2–33.2)	(30.7–34.2)
**LST Night (°C)**	Median	21.0	21.1	21.1	13.7
	(IQR)	(20.7–21.1)	(21.0–21.2)	(20.9–21.2)	(11.7–15.4)
**Elevation (m)**	Median	519	483	485	1584
	(IQR)	(506–522)	(481–486)	(482–506)	(1,446–1,768)
**Slope (°)**	Median	1.04	0.68	0.70	2.7
	(IQR)	(0.71–1.98)	(0.61–0.75)	(0.62–0.83)	(1.8–4.6)
**Rainfall (mm)**	Median	2,311	1,958	1,984	1,244
	(IQR)	(2,209–2,334)	(1,908–1,984)	(1,940–2,209)	(1,115–1,479)

Environmental variables are averaged for a buffer area of 1 km radius around each household. N = number of observations, n = number of *A*. *lumbricoides* and/or hookworm infected among *T*. *trichiura* infected in site, EPG = egg per gram of feces, SES = socio-economic status, EVI = enhanced vegetation index, LST = land surface temperature.

^a)^ According to Montresor, 1998 [27].

^b)^ Coinfection was only calculated for participants who had *T*. *trichiura* infection.

### Analysis of potential risk factors and multicollinearity

Univariable analysis for Kyela site showed that all included environmental variables were significantly associated with *T*. *trichiura* infection (Table 2). A significant inverse association was found for the potential confounders socio-economic status, population density, latrine coverage in the surroundings and presence of a latrine in the household. Being between 5 and 20 years of age was positively associated with *T*. *trichiura* infection. Previous worm treatment showed a non-significant trend towards higher prevalences in the previously treated group. Gender and household size remained non-significant and were excluded from subsequent analyses. Calculation of the variance inflation factor revealed a collinearity problem between elevation (VIF = 28.9) and rainfall (VIF = 24.7), when included into the same model. We therefore performed the multivariable analysis twice, once excluding elevation (model M1) and once excluding rainfall (model M2). Other variables did not show any serious collinearity.

**Table 2:** Univariable association of different factors with *T*. *trichiura* infection in Kyela. Results of univariable Poisson regression models adjusted for household clustering using robust variance estimates (N = 912).

Kyela both sites			Univariable^a)^		
Covariate	N	% pos.	PR	95% CI	p-value
**Age** (years)					
0–5	106	19.8	1.00	-	-
5–20	422	35.8	1.81	1.20–2.72	0.005
20 and older	384	18.5	0.93	0.59–1.47	0.766
**Worm treatment last year**					
No	565	26.9	1.00	-	-
Yes	37	37.8	1.41	0.90–2.20	0.135
No information	310	24.8	0.92	0.62–1.37	0.694
**Mean annual EVI** (per 0.1 units)			5.52	3.71–8.22	<0.001
**Mean annual rainfall** (per 100 mm)			0.49	0.41–0.59	<0.001
**Elevation** (per m)			0.91	0.88–0.95	<0.001
**Slope** (per 0.1°)			0.90	0.85–0.94	<0.001
**Mean annual LST day** (per 1°C)			0.67	0.62–0.73	<0.001
**Mean annual LST night** (per 0.1°C)			1.17	1.06–1.29	0.002
**Sex**					
Female	465	26.0	1.00	-	-
Male	447	27.3	1.05	0.85–1.30	0.665
**SES score** (per 1 unit)			0.56	0.44–0.72	<0.001
**Subsite**					
A	295	2.7	1.00	-	-
B	617	38.1	14.0	4.88–40.4	<0.001
**Household size**			1.02	0.95–1.08	0.633
**Population density** (per 1000 persons/km^2^)			0.44	0.33–0.59	<0.001
**Latrine coverage** in surroundings (per 10%)			0.75	0.63–0.88	0.001
**Household with latrine**					
No	79	39.2	1.00	-	-
Yes	833	25.5	0.65	0.43–0.98	0.041

N = number of observations in stratum, % pos. = percent *T*. *trichiura* infected in stratum, PR = prevalence ratio, 95% CI = 95% confidence interval.

^a)^ Results of separate models for each of the covariates. EVI = enhanced vegetation index, LST = land surface temperature, SES = socio-economic status.

Variables with univariable p-values below 0.2 were included in the multivariable Poisson model building. At first the individual variables, then the household level variables and as a third step the environmental variables were included in the models. Our household level model, which includes individual and household level data but no environmental data, showed that age between 5 and 20 years and previous worm treatment were risk factors for *T*. *trichiura* infection. Population density and socio-economic status were found to be protective factors (see S1 Table), whereas latrine-related variables were not included due to lack of multivariable significance.

After including environmental data, the first model for Kyela (M1, see Table 3) revealed significant positive association of *T*. *trichiura* infection with EVI (PR = 2.12, CI 1.28 to 3.50) and negative association with rainfall (PR = 0.54, CI 0.44 to 0.67) when adjusted for age and previous worm treatment. Linearity testing indicated some non-linearity of environmental variables, but fractional polynomials modelling was not carried forward, because untransformed data showed the best fit.

**Table 3:** Multivariable association of different factors with *T*. *trichiura* infection in Kyela. Results of multivariable Poisson regression models adjusted for household clustering using robust variance estimates (N = 912). Multivariable results are only shown for those variables that were included into the respective model.

Kyela both sites			Multivariable M1^a)^			Multivariable M2^b)^		
Covariate	N	% pos.	PR	95% CI	p-value	PR	95% CI	p-value
**Age** (years)								
0–5	106	19.8	1.00	-	-	1.00	-	-
5–20	422	35.8	1.98	1.38–2.83	<0.001	2.05	1.43–2.92	<0.001
20 and older	384	18.5	0.95	0.63–1.44	0.817	0.98	0.65–1.47	0.922
**Worm treatment last year**								
No	565	26.9	1.00	-	-	1.00	-	-
Yes	37	37.8	1.64	1.19–2.26	0.002	1.55	1.13–2.14	0.007
No information	310	24.8	0.99	0.71–1.37	0.953	0.88	0.64–1.20	0.421
**Mean annual EVI** (per 0.1 units)			2.12	1.28–3.50	0.003			
**Mean annual rainfall** (per 100 mm)			0.54	0.44–0.67	<0.001			
**Elevation** (per m)						0.89	0.86–0.93	<0.001
**Slope** (per 0.1°)								
**FP1 polynomial transformed slope**^c)^						0.29	0.16–0.51	<0.001
**Akaike information criterion AIC**				955			938	
**Bayesian information criterion BIC**				989			972	

N = number of observations in stratum, % pos. = percent *T*. *trichiura* infected in stratum, PR = prevalence ratio, 95% CI = 95% confidence interval, β = coefficient for fractional polynomials regression.

^a)^ Multivariable model, where elevation was excluded during the model building.

^b)^ Multivariable model where rainfall was excluded during the model building. EVI = enhanced vegetation index.

^c)^ FP1 fractional polynomial transformation with one degree and power of p = -1: β(slope) ^-1^.

The second model for Kyela (M2, see Table 3) showed significant negative associations of *T*. *trichiura* infection with elevation (PR = 0.89, CI 0.86 to 0.93) and transformed slope while adjusting for age and previous worm treatment. The slope was modelled as a fractional polynomial of degree one (FP1) with power p = -1 yielding the equation β*(slope) ^-1^ = β/slope. Exponentiation of the coefficient β (= -1.25) results in a prevalence ratio of 0.29 (CI 0.16 to 0.51) which corresponds to a positive association of *T*. *trichiura* infection with increasing slope. In addition to this, we included minimal LST during the night and maximal LST during the day into our model building. None of the two LST variables stayed significant in the multivariable models.

The potential confounders socio-economic status and population density do not seem to confound the associations between *T*. *trichiura* infection and environmental variables, since they turned non-significant after inclusion of environmental variables. Latrine-related variables lost their significance already in the household level model and thus also did not confound this association.

Including a dummy variable for subsite (Kyela A vs. Kyela B) in the final models did not improve the model (see S2 Table), either. Running the models for Kyela A and Kyela B separately confirmed above results for Kyela B (see S3 Table). This is reasonable since due to larger sample size and higher prevalence, the influence of Kyela B in the complete model is much larger than the influence of the other subsite. For Kyela A the model could not be run due to low cell counts (of the eight infections found in Kyela A none was in the reference age group of 0 to 5 years).

**Fig 4:**
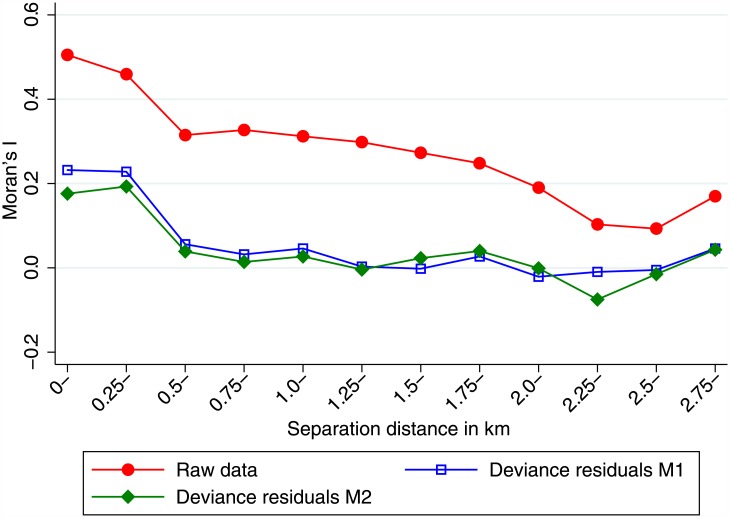
Spatial autocorrelation of *T*. *trichiura* infection between and within households in Kyela. The red line shows Moran’s I of spatial autocorrelation for the raw data. The blue and green lines show the autocorrelation of deviance residuals for the models M1 and M2, respectively. The horizontal axis shows the distance bands between households.

The spatial autocorrelation, that was present in the raw data, was strongly reduced in the deviance residuals of both multivariable models (Fig 4) that are shown in Table 3. This indicates that the models account for a large part of the spatial autocorrelation in the raw data.

## Discussion

Our results show that *T*. *trichiura* infection in Kyela site is significantly associated with environmental variables in both uni- and multivariable analysis. Participant age of 5 to 20 years, previous worm treatment and green vegetation showed significant positive associations and were thus retained in the multivariable models. Inverse uni- and multivariable associations were found for rainfall and elevation. Slope of the terrain was modelled non-linearly corresponding to a positive association of infections with increasing slope.

Since we found only six *T*. *trichiura* infections in the other eight study sites we were unsure whether these infections were imported or really acquired locally. We therefore only included data from Kyela site into regression analyses. Even in Kyela the prevalence difference between both subsites was remarkable (2.7% vs. 38.1%, see Table 1).

The association of infection prevalence with green vegetation seems reasonable, since vegetation provides shade, which protects eggs from ultraviolet radiation and desiccation and also serves as a proxy for soil moisture, which is needed for the development of *T*. *trichiura* eggs. Most studies do not assess vegetation as a potential risk factor for *T*. *trichiura*. However, since both *A*. *lumbricoides* and *T*. *trichiura* infections are transmitted by ingesting eggs, which develop in the soil [28], the finding of a positive association of *A*. *lumbricoides* infection with denser vegetation [29, 30] might also apply for *T*. *trichiura*.

The inverse association of *T*. *trichiura* infection with rainfall also makes sense, because the mean annual rainfall in Kyela is the highest among all EMINI study sites. For the existence and development of living organisms, such as *T*. *trichiura* eggs in our case, there are optimal environmental conditions. Before reaching the optimum condition the variable associated with the infection acts as risk factor, beyond the optimum it has a protective character. Our association of *T*. *trichiura* infection with rainfall reflects the latter case. Indeed, in the subsite Kyela B some areas are regularly flooded, a condition presumably prohibiting the eggs from developing to the infective stage. This might also explain why in our analysis slope was a risk factor for infection. Water bodies on the ground are located on a flat surface. Slope thus means that the terrain is usually not flooded. However, the association of *T*. *trichiura* infection with rainfall depends on the setting. As in our study, Pullan *et al*. [31] found a trend towards an inverse association between infection and rainfall, whereas Scholte *et al*. [32] found rainfall to be a risk factor for *T*. *trichiura* infection.

A decrease in *T*. *trichiura* infection prevalence with increasing elevation was also found by other authors [30–33]. This effect is likely due to decreasing temperature at higher altitudes, which might negatively affect the development of *T*. *trichiura* eggs. In Kyela site, this strong negative association is already visible on a per meter scale, which is remarkable. The nearly complete absence of *T*. *trichiura* infection in other sites might thus be a consequence of the higher elevation, and, in turn, lower temperatures, of these other sites, although this is difficult to judge from our data.

The univariable inverse association of LST day with infection is consistent with previous research [31, 32]. The likely explanation is that higher soil temperatures lead to desiccation of the soil and the parasite eggs. Furthermore high temperature could directly damage the eggs: a laboratory study found that the development of *T*. *trichiura* eggs ceases at 37°C [8]. A similar finding for combined data from Sub-Saharan Africa was reported by Pullan et al. [31]. Our LST day variable lost its significance in the multivariable modelling, since other variables better predicted *T*. *trichiura* prevalence. LST night was found to be a risk factor, a finding plausible with the above-mentioned optimum conditions for *T*. *trichiura* eggs’ development. The relatively low night temperatures are below the optimum and thus higher temperatures promote development and so act as a risk factor for the infection.

The non-linear association of infection prevalence with slope was best modelled using a fractional polynomial, whereas all other factors could be modelled using untransformed data. Within household clustering of infection was taken into account by using robust variance estimates. As Fig 3 shows, the spatial autocorrelation in the final models was reduced, which means that the variables included in the multivariable models accounted for a considerable part of the spatial clustering. The remaining autocorrelation might lead to slightly lower estimates of variances and p-values, although we do not think that this effect is very strong.

It seems a common finding that *T*. *trichiura* infection is higher close to water bodies [30–32, 34]. This could be related to low altitude, moderated temperatures and high soil moisture near water. In our study, the spatial distribution of *T*. *trichiura* infection was limited to the vicinity of Lake Nyasa in Kyela. The affinity to water was not only found in Sub-Saharan Africa [30, 31, 34], but also in the Americas [32].

Sensitivity analysis that additionally included subsite as a binary variable in the final models (see S2 Table) showed that most of the prevalence difference between subsites was explained by our measured variables.

The positive association of *T*. *trichiura* with previous worm treatment is counter-intuitive, but given the frequently observed high reinfection rate [35] and the low effectiveness of the available drugs against *T*. *trichiura* infection [5, 6], our results seem reasonable. Seeking worm treatment in the first place suggests that the participants had, or at least assumed to have a helminth infection. This in turn might speak for a high exposure to the risk factors for helminth infections or simply for living in a place with favorable conditions for helminths, which in our study is supported by the fact that the majority of *T*. *trichiura* infected participants were co-infected with *A*. *lumbricoides* and/or hookworm (see Table 1). Finally, *T*. *trichiura* is relatively difficult to treat, so that previous deworming, even if it happened recently, does not necessarily mean that *T*. *trichiura* infection has been cured [36].

The finding that *T*. *trichiura* infection is more prevalent in children and adolescents than in adults (see Fig 3) is consistent with the epidemiology of these infections according to the literature [1]. The higher prevalence can be related to behavioral aspects like playing outside with lots of soil contact and not yet having fully adopted hygiene habits. The second prevalence peak between 50 and 80 years of age has not yet been reported in the literature. It is caused by only few infected individuals and might be a chance finding, or be due to some unmeasured characteristic of our study population.

Our results revealed mostly large-scale associations of environmental variables with *T*. *trichiura* infection. In the final models the household-level factors population density and socio-economic status turned non-significant, indicating that in our study population environmental conditions are better predictors than the household-level factors. The same is true for latrine ownership and for latrine coverage in the household surroundings: although significant when considered on their own, both variables turn non-significant when included in a multivariable model together with the ecological factors. However, the individual factors age and previous worm treatment remain significant in all models. This speaks for the usage of remotely sensed large-scale environmental data to predict risk or prevalence of soil-transmitted helminth infections, but also recognizes the importance to assess individual disease-related variables.

### Study limitations and strengths

We restricted the main part of our analysis to Kyela, since only six infections were found in the other eight study sites. It is not completely clear whether these infections where acquired elsewhere and thus imported or if they indicate local transmission within the study sites. Generalizing the results to other study sites and regions should thus be done with caution.

Our cross-sectional study-design is unable to assess incidence of *T*. *trichiura* infection and changes over time. To our knowledge, no mass deworming programs had been conducted in the study region allowing us to capture the natural prevalence, which was not affected by any running programs. Although we asked for individual worm treatment history during interviews, many of these data were missing. In Kyela one third of the worm treatment data were missing, but the stratum with missing data did not show an association with infection. The Kato-Katz method, which was used to diagnose *T*. *trichiura* infection, is relatively insensitive especially in light infections with low egg-counts, which could have led to underestimates of prevalence.

One strength of our study is the large sample size. The study sites represent a wide variety of environmental and ecological conditions. We had individual, household and environmental data to investigate small scale and larger scale associations. We did extensive modelling, tested for non-linearity, multicollinearity and used fractional polynomials to confirm the robustness of our models.

For future studies, it might be of interest to investigate the link of *T*. *trichiura* infection with areas near water specifically. To find out which type of soil is most suitable for *T*. *trichiura* eggs’ development, the soil type and texture at each household would also be of interest, but unfortunately this information was not available for our study area.

### Conclusion

We found a unique maximum of *T*. *trichiura* infection prevalence in a study site characterized by flat terrain, low elevation and high amounts of green vegetation and rain, which is situated close to Lake Nyasa. *T*. *trichiura* infection was associated with child- and adolescent-age, previous treatment for helminth infection, amount of vegetation, slope, low rainfall and low elevation. This shows that *T*. *trichiura* infection is strongly linked to environmental factors, which could thus be used to predict high-risk areas for targeted helminth control.

## Supporting information

S1 File STROBE checklist. (DOCX)

S1 Table Household level model for *T*. *trichiura* infection in Kyela. Results of multivariable Poisson regression adjusted for household clustering using robust variance estimates (N = 912).
(DOCX)

S2 Table Influence of subsite variable on the final models M1 and M2. Results of multivariable Poisson regressions adjusted for household clustering using robust variance estimates for Kyela (N = 912).
(DOCX)

S3 Table Final models including only participant data from subsite Kyela B. Results of multivariable Poisson regressions adjusted for household clustering using robust variance estimates for Kyela B (N = 617).
(DOCX)
