# Supplementary material for: Trichuris trichiura infection and its relation to environmental factors in Mbeya region, Tanzania: A cross-sectional, population-based study
Source: PLoS One. 2017 Apr 6;12(4):e0175137. doi: 10.1371/journal.pone.0175137 (PMC5383155; doi:10.1371/journal.pone.0175137)
Supplement: S1 File — (DOCX) [file pone.0175137.s001.docx]

### **File S4 -STROBE checklist**

STROBE Statement—Checklist of items that should be included in reports of ***cross-sectional studies***

|  | **Item No** | **Recommendation** | **Relevant text from Manuscript** |
| --- | --- | --- | --- |
| **Title and abstract** | 1 | (*a*) Indicate the study’s design with a commonly used term in the title or the abstract | *Trichuris trichiura* Infection and Its Relation to Environmental Factors in Mbeya Region, Tanzania: A Cross-Sectional, Population-Based Study **[p. 1]** |
|  |  | (*b*) Provide in the abstract an informative and balanced summary of what was done and what was found | We analyzed data from a cross-sectional survey including 6234 participants from nine distinct study sites in Mbeya region, Tanzania. A geographic information system was used to combine remotely sensed and individual data **[p.2]**  *T. trichiura* infection was restricted to the Kyela site, close to Lake Nyasa with only very few cases in the other eight sites. The prevalence of *T. trichiura* infection in Kyela was 26.6% (95% confidence interval (CI) 23.9 to 29.6%). Multivariable models revealed a positive association of infection with denser vegetation (prevalence ratio (PR) per 0.1 EVI units = 2.12, CI 1.28 to 3.50) and inverse associations with rainfall (PR per 100 mm = 0.54, CI 0.44 to 0.67) and elevation (PR per meter = 0.89, CI 0.86 to 0.93) while adjusting for age and previous worm treatment. Slope of the terrain was modelled non-linearly and also showed a positive association with *T. trichiura* infection (p-value p<0.001). **[p. 2]** |
| **Introduction** | | |  |
| Background/  rationale | 2 | Explain the scientific background and rationale for the investigation being reported | …*T. trichiura* accounts for about 465 million infections world-wide [2]. Sub-Saharan Africa is one of the regions still heavily affected by soil-transmitted helminth infections, since their transmission is enhanced by poor hygienic conditions and poverty. Unfortunately, despite the efforts of preventive mass chemotherapy conducted in many sub-Saharan African countries [3], the *T. trichiura* prevalence there has not recently declined [2, 4]. Indeed, *T. trichiura* infection seems to be difficult to cure, since the available drugs are not very effective against this helminth infection [5, 6]. **[p. 3-4]**  No prevalence estimates for *T. trichiura* infection in southwestern Tanzania are available from the literature [12]. This study provides new information on the prevalence and the spatial distribution of *T. trichiura* infection from an epidemiological survey conducted in Mbeya region in southwestern Tanzania. **[p. 4]** |
| Objectives | 3 | State specific objectives, including any prespecified hypotheses | Our aim was to investigate the associations of satellite derived environmental data with *T. trichiura* infection while considering the effect of potential confounders, such as age, sex and socio-economic status. **[p. 5]** |
| **Methods** | | |  |
| Study design | 4 | Present key elements of study design early in the paper | Initially nine different study sites in Mbeya region were chosen to represent a wide variety of environmental and economic conditions. After an initial census covering more than 42,000 households from these nine sites, a geographically stratified random sample of 10% of the households was chosen to participate in the EMINI study. **[p. 5]**  See also Item No 5 below. |
| Setting | 5 | Describe the setting, locations, and relevant dates, including periods of recruitment, exposure, follow-up, and data collection | The study area is located in the Mbeya region in southwestern Tanzania. The data was collected between June 2008 and June 2009 as a part of the third annual survey of the EMINI (Evaluating and Monitoring the Impact of New Interventions) cohort study. Initially nine different study sites in Mbeya region were chosen to represent a wide variety of environmental and economic conditions. **[p. 5]**  Each household’s position was determined using handheld GPS devices. During each annual visit, all household members were asked for blood and urine samples and interviewed in Kiswahili language and their answers recorded using handheld computers. Additionally, we collected stool samples from the third annual survey in 2008 onwards from 50% of all households. **[p. 5]** |
| Participants | 6 | (*a*) Give the eligibility criteria, and the sources and methods of selection of participants | After an initial census covering more than 42,000 households from these nine sites, a geographically stratified random sample of 10% of the households was chosen to participate in the EMINI study. **[p. 4]**  Additionally, stool samples were collected from the third annual survey in 2008 onwards from 50% of all households. **[p. 4]** |
| Variables | 7 | Clearly define all outcomes, exposures, predictors, potential confounders, and effect modifiers. Give diagnostic criteria, if applicable | Due to only light to moderate *T. trichiura* infection intensity and thus low egg counts we modelled the infection outcome as a binary variable (negative vs. positive). **[p. 7]**  Our aim was to investigate the associations of satellite derived environmental data with *T. trichiura* infection. **[p. 5]**  Socio-economic status (SES) can be a potential confounder and should thus be assessed. **[p. 6]**  Age, sex, socio-economic status, population density, latrine coverage in the surroundings, presence of a latrine in the household and previous worm treatment were included into our analyses as potential confounders. **[p. 7]**  The presence of *T. trichiura* infection was established by Kato-Katz examination of two 41.7 mg subsamples from a single stool specimen and defined as existence of at least one *T. trichiura* egg in any of the two stool slides. **[p. 6]** |
| Data sources/ measurement | 8* | For each variable of interest, give sources of data and details of methods of assessment (measurement). Describe comparability of assessment methods if there is more than one group | During annual visits all household members were interviewed in Kiswahili language and their answers recorded using handheld computers. During each household visit blood and urine samples were collected. Additionally, stool samples were collected from the third annual survey in 2008 onwards from 50% of all households. **[p. 5]**  The presence of *T. trichiura* infection was established by Kato-Katz examination of two 41.7 mg subsamples from a single stool specimen and defined as existence of at least one *T. trichiura* egg in any of the two stool slides. **[p. 6]**  For our study, the SES score was constructed from household belongings (clock or watch, radio, television, mobile telephone, refrigerator, hand cart, bicycle, motor cycle, car, savings account), materials used to build the house, sources of energy and drinking water, number of persons per room and availability and type of latrine. This information was obtained from the head of the household and by direct observation during the interviews. **[p. 6]**  Elevation data for our study were retrieved from the NASA Shuttle Radar Topography Mission (SRTM) global digital elevation model, version 2.1. [16]. These data were also used to calculate the slope of the terrain. Land surface temperature during the day (LST day) and night (LST night), and green vegetation cover (EVI = enhanced vegetation index) data were collected from NASA’s Moderate-Resolution Imaging Spectroradiometer (MODIS) Terra satellite during 2003 to 2008. These data were retrieved from the online data pool, courtesy of the NASA EOSDIS Land Processes Distributed Active Archive Center (LP DAAC), USGS/Earth Resources Observation and Science (EROS) Center, Sioux Falls, South Dakota (https://lpdaac.usgs.gov/) [17] and were used to produce long-term averages of LST day, LST night and EVI, as previously described [13]. Mean annual rainfall was obtained from the WorldClim – Global Climate Data website (<http://www.worldclim.org/>) **[p. 6-7]**  Household positions and number of inhabitants were known from the initial population census and used to calculate population densities. **[p. 7]** |
| Bias | 9 | Describe any efforts to address potential sources of bias | The presence of *T. trichiura* infection was established by Kato-Katz examination of two 41.7 mg subsamples from a single stool specimen and defined as existence of at least one *T. trichiura* egg in any of the two stool slides. **[p. 6]**  The population density, LST, EVI, rainfall and elevation data were then averaged for a buffer area of 1000 m radius around each household to characterize the situation around the household. **[p. 7]** |
| Study size | 10 | Explain how the study size was arrived at | Please see Item No. 6 above. |
| Quantitative variables | 11 | Explain how quantitative variables were handled in the analyses. If applicable, describe which groupings were chosen and why | We applied the following variable transformations to improve interpretability of results: the reported prevalence ratios (PRs) correspond to an increase of 1000 persons/km^2^ for the population density, 0.1 units for mean annual EVI, 0.1 °C for mean annual LST night, 100 mm for rainfall and 10% for latrine prevalence in the surroundings. Age was categorized in groups of 0 to 5 years, 5 to 20 years and above 20 years, since a non-linear relationship of *T. trichiura* infection and age was expected from the literature [1]. **[p. 7-8]**  Calculation of the variance inflation factor revealed a collinearity problem between elevation (VIF = 28.9) and rainfall (VIF = 24.7), when included into the same model. We therefore performed the multivariable analysis twice, once excluding elevation (model M1) and once excluding rainfall (model M2). **[p. 13]** |
| Statistical methods | 12 | (*a*) Describe all statistical methods, including those used to control for confounding | … multicollinearity of independent variables was assessed using the variance inflation factor (VIF). **[p. 7]**  To assess the association of different factors with *T. trichiura* infection we used Poisson regression with robust (or Huber/White or Sandwich) variance estimates **[p. 8]**  … our data is clustered within households. To account for this robust standard errors adjusted for household clustering were calculated. After each univariable analysis step we performed a non-linearity test for the respective independent variable [23]. **[p. 8]**  All variables with univariable p-values below 0.2 were initially included in the multivariable analysis. Multivariable Poisson regression was performed by starting with a model including only individual level covariates such as sex, age and worm treatment history. The model was extended by including household level covariates such as availability of latrine or socio-economic status. As a third step, we included environmental variables one by one. Variables were retained in the model if their p-values stayed <= 0.05 and variables with p-values above 0.05 were removed. **[p. 8]**  Because of the non-linearity of the environmental data, we also used fractional polynomials modelling … **[p. 8]**  To assess the existence and degree of spatial autocorrelation, Moran’s I [26] was calculated for different distances between household positions. **[p. 9]** |
|  |  | (*b*) Describe any methods used to examine subgroups and interactions | Including a dummy variable for subsite (Kyela A vs. Kyela B) in the final models did not improve the model (see supplementary table S2), either. Running the models for Kyela A and Kyela B separately confirmed above results for Kyela B (see supplementary table S3). **[p. 17]**  Interactions are not considered meaningful for our analysis, since the models consist of environmental variables, which by nature are prone to be multicollinear with each other. |
|  |  | (*c*) Explain how missing data were addressed | Please see the stratum “no information” in the worm treatment variable in Tables 1, 2 and 3. **[p. 12, 14, 16]**  Although we asked for individual worm treatment history during interviews, many of these data were missing. In Kyela one third of the worm treatment data were missing, but the stratum with missing data did not show an association with infection. **[p. 22]** |
|  |  | (*d*) If applicable, describe analytical methods taking account of sampling strategy | n/a |
|  |  | (*e*) Describe any sensitivity analyses | Since environmental data is prone to be highly correlated, multicollinearity of independent variables was assessed using the variance inflation factor (VIF). **[p. 7]**  After each univariable analysis step we performed a non-linearity test for the respective independent variable [23]. **[p. 8]**  Because of the non-linearity of the environmental data, we also used fractional polynomials modelling to check, if the multivariable linear Poisson models required inclusion of non-linear terms. **[p. 8]** |
| **Results** | | |  |
| Participants | 13* | (a) Report numbers of individuals at each stage of study—eg numbers potentially eligible, examined for eligibility, confirmed eligible, included in the study, completing follow-up, and analysed | Not considered necessary. |
|  |  | (b) Give reasons for non-participation at each stage | Not considered necessary. |
|  |  | (c) Consider use of a flow diagram | Not considered necessary. |
| Descriptive data | 14* | (a) Give characteristics of study participants (eg demographic, clinical, social) and information on exposures and potential confounders | Please see Table 1. **[p. 12]** |
|  |  | (b) Indicate number of participants with missing data for each variable of interest | Please see the stratum “no information” in the worm treatment variable in Tables 1, 2 and 2. **[p. 12, 14, 16]** |
| Outcome data | 15* | Report numbers of outcome events or summary measures | The overall prevalence of *T. trichiura* infection in all nine sites was 4.0% (249/6234, 95% confidence interval (CI) 3.5 to 4.5%). We found a unique local prevalence maximum of 26.6% (243/912, CI 23.9 to 29.6%) in Kyela study site (Table 1). **[p. 9]** |
| Main results | 16 | (*a*) Give unadjusted estimates and, if applicable, confounder-adjusted estimates and their precision (eg, 95% confidence interval). Make clear which confounders were adjusted for and why they were included | For unadjusted estimates please see column “Univariable” in Table 2. **[p. 14]**  After including environmental data, the first model for Kyela (M1, see Table 3) revealed significant positive association of *T. trichiura* infection with EVI (PR = 2.12, CI 1.28 to 3.50) and negative association with rainfall (PR = 0.54, CI 0.44 to 0.67) when adjusted for age and previous worm treatment. **[p. 15]**  The second model for Kyela (M2, see Table 3) showed significant negative associations of *T. trichiura* infection with elevation (PR = 0.89, CI 0.86 to 0.93) and transformed slope while adjusting for age and previous worm treatment. The slope was modelled as a fractional polynomial of degree one (FP1) with power p=-1 yielding the equation β*(slope) ^-1^ = β/slope. Exponentiation of the coefficient β (= -1.25) results in a prevalence ratio of 0.29 (CI 0.16 to 0.51) which corresponds to a positive association of *T. trichiura* infection with increasing slope. **[p. 16]** |
|  |  | (*b*) Report category boundaries when continuous variables were categorized | Age was categorized in groups of 0 to 5 years, 5 to 20 years and above 20 years, since a non-linear relationship of *T. trichiura* infection and age was expected from the literature [1]. **[p. 7-8]** |
|  |  | (*c*) If relevant, consider translating estimates of relative risk into absolute risk for a meaningful time period | Considered not relevant. |
| Other analyses | 17 | Report other analyses done—eg analyses of subgroups and interactions, and sensitivity analyses | The slope was modelled as a fractional polynomial of degree one (FP1) with power p=-1 yielding the equation β*(slope) ^-1^ = β/slope. Exponentiation of the coefficient β (= -1.25) results in a prevalence ratio of 0.29 (CI 0.16 to 0.51) which corresponds to a positive association of *T. trichiura* infection with increasing slope. **[p. 16]**  Including a dummy variable for subsite (Kyela A vs. Kyela B) in the final models did not improve the model (see supplementary table S2), either. Running the models for Kyela A and Kyela B separately confirmed above results for Kyela B (see supplementary table S3). **[p. 17]**  The spatial autocorrelation, that was present in the raw data, was strongly reduced in the deviance residuals of both multivariable models (Fig. 3) that are shown in Table 3. This indicates that the models account for a large part of the spatial autocorrelation in the raw data. **[p. 17]** |
| **Discussion** | | |  |
| Key results | 18 | Summarise key results with reference to study objectives | Our results show that *T. trichiura* infection in Kyela site is significantly associated with environmental variables in both uni- and multivariable analysis. Participant age of 5 to 20 years, previous worm treatment and green vegetation showed significant positive associations and were thus retained in the multivariable models. Inverse uni- and multivariable associations were found for rainfall and elevation. Slope of the terrain was modelled non-linearly corresponding to a positive association of infections with increasing slope. **[p. 18]** |
| Limitations | 19 | Discuss limitations of the study, taking into account sources of potential bias or imprecision. Discuss both direction and magnitude of any potential bias | We restricted the main part of our analysis to Kyela, since only six infections were found in the other eight study sites. It is not completely clear whether these infections where acquired elsewhere and thus imported or if they indicate local transmission within the study sites. Generalizing the results to other study sites and regions should thus be done with caution. **[p. 22]**  Our cross-sectional study-design is unable to assess incidence of *T. trichiura* infection and changes over time. **[p. 22]**  Although individual worm treatment history was asked for during interviews, many of these data were missing. In Kyela one third of the worm treatment data were missing, but the stratum with missing data did not show an association with infection. **[p. 22]**  The Kato-Katz method, which was used to diagnose *T. trichiura* infection, is relatively insensitive especially in light infections with low egg-counts, which could lead to underestimates of prevalence. **[p. 22]** |
| Interpretation | 20 | Give a cautious overall interpretation of results considering objectives, limitations, multiplicity of analyses, results from similar studies, and other relevant evidence | Our results revealed mostly large-scale associations of environmental variables with *T. trichiura* infection. In the final models the household-level factors population density and socio-economic status turned non-significant, indicating that in our study population environmental conditions are better predictors than the household-level factors. The same is true for latrine ownership and for latrine coverage in the household surroundings: although significant when considered on their own, both variables turn non-significant when included in a multivariable model together with the ecological factors. However, the individual factors age and previous worm treatment remain significant in all models. This speaks for the usage of remotely sensed large-scale environmental data to predict risk or prevalence of soil-transmitted helminth infections, but also recognizes the importance to assess individual disease-related variables. **[p. 21-22]** |
| Generalisability | 21 | Discuss the generalisability (external validity) of the study results | Generalizing the results to other study sites and regions should thus be done with caution. **[p. 22]** |
| **Other information** | | |  |
| Funding | 22 | Give the source of funding and the role of the funders for the present study and, if applicable, for the original study on which the present article is based | The EMINI study was funded by the European Commission (SANTE/2004/078-545/130 & SANTE/2006/129-931). Helminth data collection was supported by the German Science foundation DFG (SA 1878/1-1) and the German Federal Ministry of Education and Research BMBF (01 KA 0904) with additional support from the European Commision’s Seventh Framework Programme (EC-GA n^o^ 241642). The funders had no role in study design, data collection and analysis, decision to publish, or preparation of the manuscript. |

*Give information separately for exposed and unexposed groups.
