# Supplementary material for: Trichuris trichiura infection and its relation to environmental factors in Mbeya region, Tanzania: A cross-sectional, population-based study
Source: PLoS One. 2017 Apr 6;12(4):e0175137. doi: 10.1371/journal.pone.0175137 (PMC5383155; doi:10.1371/journal.pone.0175137)
Supplement: S1 Table — Results of multivariable Poisson regression adjusted for household clustering using robust variance estimates (N = 912). (DOCX) [file pone.0175137.s002.docx]

**Table S1: Household level model for *T. trichiura* infection in Kyela.** Results of multivariable Poisson regression adjusted for household clustering using robust variance estimates (N=912).

| Kyela both sites |  |  | Household level model^a)^ | | |
| --- | --- | --- | --- | --- | --- |
| Covariate | N | % pos. | PR | 95% CI | p-value |
| **Age** (years) |  |  |  |  |  |
| 0-5 | 106 | 19.8 | 1.00 | - | - |
| 5-20 | 422 | 35.8 | 1.95 | 1.31 – 2.90 | 0.001 |
| 20 and older | 384 | 18.5 | 1.02 | 0.65 – 1.60 | 0.920 |
| **Worm treatment last year** |  |  |  |  |  |
| No | 565 | 26.9 | 1.00 | - | - |
| Yes | 37 | 37.8 | 1.66 | 1.04 – 2.66 | 0.035 |
| No information | 310 | 24.8 | 1.05 | 0.71 – 1.55 | 0.819 |
| **SES score** (per 1 unit) |  |  | 0.65 | 0.50 – 0.86 | 0.002 |
| **Population density** (per 1000 persons/km^2^) |  |  | 0.56 | 0.41 – 0.77 | <0.001 |
| **Akaike information criterion AIC** |  |  |  | 1060 |  |
| **Bayesian information criterion BIC** |  |  |  | 1094 |  |
| N = number of observations in stratum, % pos. = percent *T. trichiura* infected in stratum, PR = prevalence ratio, 95% CI = 95% confidence interval. ^a)^ Multivariable model without inclusion of environmental variables. SES = socio-economic status. | | | | | |
