# Supplementary material for: Trichuris trichiura infection and its relation to environmental factors in Mbeya region, Tanzania: A cross-sectional, population-based study
Source: PLoS One. 2017 Apr 6;12(4):e0175137. doi: 10.1371/journal.pone.0175137 (PMC5383155; doi:10.1371/journal.pone.0175137)
Supplement: S2 Table — Results of multivariable Poisson regressions adjusted for household clustering using robust variance estimates for Kyela (N = 912). (DOCX) [file pone.0175137.s003.docx]

**Table S2: Influence of subsite variable on the final models M1 and M2.** Results of multivariable Poisson regressions adjusted for household clustering using robust variance estimates for Kyela (N=912).

| Kyela both sites |  |  | Multivariable M1 with subsite^a)^ | | | Multivariable M2 with subsite^b)^ | | |
| --- | --- | --- | --- | --- | --- | --- | --- | --- |
| Covariate | N | % pos. | PR | 95% CI | p-value | PR | 95% CI | p-value |
| **Age** (years) |  |  |  |  |  |  |  |  |
| 0-5 | 106 | 19.8 | 1.00 | - | - | 1.00 | - | - |
| 5-20 | 422 | 35.8 | 2.02 | 1.41 – 2.89 | <0.001 | 2.02 | 1.42 – 2.90 | <0.001 |
| 20 and older | 384 | 18.5 | 0.97 | 0.64 – 1.47 | 0.883 | 0.97 | 0.65 – 1.46 | 0.892 |
| **Worm treatment last year** |  |  |  |  |  |  |  |  |
| No | 565 | 26.9 | 1.00 | - | - | 1.00 | - | - |
| Yes | 37 | 37.8 | 1.65 | 1.20 – 2.27 | 0.002 | 1.53 | 1.11 – 2.12 | 0.010 |
| No information | 319 | 24.5 | 0.96 | 0.65 – 1.34 | 0.830 | 0.88 | 0.65 – 1.21 | 0.436 |
| **Subsite** |  |  |  |  |  |  |  |  |
| A | 295 | 2.7 | 1.00 | - | - | 1.00 | - | - |
| B | 617 | 38.1 | 2.38 | 0.68 – 8.35 | 0.174 | 0.33 | 0.07 – 1.66 | 0.179 |
| **Mean annual EVI** (per 0.1 units) |  |  | 1.84 | 1.08 – 3.15 | 0.026 |  |  |  |
| **Mean annual rainfall** (per 100 mm) |  |  | 0.62 | 0.48 – 0.80 | <0.001 |  |  |  |
| **Elevation** (per m) |  |  |  |  |  | 0.87 | 0.82 – 0.92 | <0.001 |
| **FP1 polynomial transformed slope**^c)^ |  |  |  |  |  | 0.23 | 0.11 – 0.48 | <0.001 |
| **Akaike information criterion AIC** |  |  |  | 954 |  |  | 938 |  |
| **Bayesian information criterion BIC** |  |  |  | 993 |  |  | 977 |  |
| N = number of observations in stratum, % pos. = percent *T. trichiura* infected in stratum, PR = prevalence ratio, 95% CI = 95% confidence interval. ^a)^ Multivariable model including only age, previous worm treatment, subsite, mean annual EVI and rainfall but not elevation, which was excluded because of collinearity with rainfall. ^b)^ Multivariable model including only age, previous worm treatment, subsite, elevation and slope of the terrain but not rainfall which was excluded because of collinearity with elevation. EVI = enhanced vegetation index. ^c)^ FP1 fractional polynomial transformation with one degree and power of p=-1: β(slope)^p^. | | | | | | | | |
