# Supplementary material for: Trichuris trichiura infection and its relation to environmental factors in Mbeya region, Tanzania: A cross-sectional, population-based study
Source: PLoS One. 2017 Apr 6;12(4):e0175137. doi: 10.1371/journal.pone.0175137 (PMC5383155; doi:10.1371/journal.pone.0175137)
Supplement: S3 Table — Results of multivariable Poisson regressions adjusted for household clustering using robust variance estimates for Kyela B (N = 617). (DOCX) [file pone.0175137.s004.docx]

**Table S3: Final models including only participant data from subsite Kyela B.** Results of multivariable Poisson regressions adjusted for household clustering using robust variance estimates for Kyela B (N=617).

| Subsite Kyela B |  |  | Multivariable M1^a)^ | | | Multivariable M2^b)^ | | |
| --- | --- | --- | --- | --- | --- | --- | --- | --- |
| Covariate | N | % pos. | PR | 95% CI | p-value | PR | 95% CI | p-value |
| **Age** (years) |  |  |  |  |  |  |  |  |
| 0-5 | 74 | 28.4 | 1.00 | - | - | 1.00 | - | - |
| 5-20 | 271 | 53.5 | 1.97 | 1.38 – 2.81 | <0.001 | 2.04 | 1.42 – 2.92 | <0.001 |
| 20 and older | 272 | 25.4 | 0.95 | 0.62 – 1.44 | 0.796 | 0.97 | 0.64 – 1.47 | 0.901 |
| **Worm treatment last year** |  |  |  |  |  |  |  |  |
| No | 374 | 38.5 | 1.00 | - | - | 1.00 | - | - |
| Yes | 25 | 56.0 | 1.74 | 1.27 – 2.38 | 0.001 | 1.61 | 1.17 – 2.22 | 0.004 |
| No information | 218 | 35.3 | 1.03 | 0.74 – 1.42 | 0.876 | 0.96 | 0.71 – 1.31 | 0.818 |
| **Mean annual EVI** (per 0.1 units) |  |  | 1.74 | 1.03 – 2.93 | 0.038 |  |  |  |
| **Mean annual rainfall** (per 100 mm) |  |  | 0.59 | 0.45 – 0.77 | <0.001 |  |  |  |
| **Elevation** (per m) |  |  |  |  |  | 0.86 | 0.81 – 0.91 | <0.001 |
| **FP1 polynomial transformed slope**^c)^ |  |  |  |  |  | 0.32 | 0.16 – 0.61 | 0.001 |
| **Akaike information criterion AIC** |  |  |  | 880 |  |  | 868 |  |
| **Bayesian information criterion BIC** |  |  |  | 911 |  |  | 899 |  |
| N = number of observations in stratum, % pos. = percent *T. trichiura* infected in stratum, PR = prevalence ratio, 95% CI = 95% confidence interval. ^a)^ Multivariable model including only age, previous worm treatment, mean annual EVI and rainfall. ^b)^ Multivariable model including only age, previous worm treatment, elevation and slope of the terrain. EVI = enhanced vegetation index. ^c)^ FP1 fractional polynomial transformation with one degree and power of p=-1: β(slope)^p^. | | | | | | | | |

Note: The models M1 and M2 cannot be estimated for Kyela A, since there are no infected participants in the reference age category of 0 to 5 years.
